# Complementarity in Phenolic Compounds and the Antioxidant Activities of *Phaseolus coccineus* L. and *P. vulgaris* L. Landraces

**DOI:** 10.3390/foods8080295

**Published:** 2019-07-28

**Authors:** Arelly Capistrán-Carabarin, Elia Nora Aquino-Bolaños, Yatzil Denih García-Díaz, José Luis Chávez-Servia, Araceli Minerva Vera-Guzmán, José Cruz Carrillo-Rodríguez

**Affiliations:** 1 Instituto de Ciencias Básicas, Universidad Veracruzana, Xalapa, Veracruz 91194, Mexico; 2 CIIDIR-Oaxaca, Instituto Politécnico Nacional, Santa Cruz Xoxocotlán, Oaxaca 71230, Mexico; 3 Instituto Tecnológico del Valle de Oaxaca, Santa Cruz Xoxocotlán, Oaxaca 71230, Mexico

**Keywords:** scarlet runner bean, spectrophotometry, bioactive compounds, landraces, indigenous communities

## Abstract

*Phaseolus vulgaris* L. is one of the most consumed and documented legumes in regard to its grain composition, but little is known about *P. coccineus* L. To evaluate and compare the phenolic compound content and antioxidant activity between landraces of *P. coccineus* and *P. vulgaris*, a total of 14 accessions of *P. coccineus* and *P. vulgaris* were collected from farmers in Oaxaca, Mexico. Based on reference standards and spectrophotometry, the polyphenol, flavonoid and anthocyanin contents were quantified, and the antioxidant activity was determined by the 2,2-diphenyl-1-picrylhydrazyl (DPPH) method. The results showed significant differences (*p* ≤ 0.05) between species and accessions, where *P. coccineus* and *P. vulgaris* significantly differed in their contents of polyphenols, flavonoids, and anthocyanins, as well as their antioxidant activity in the seed coat and cotyledons. Higher concentrations were found in the seed coat than in the cotyledons for both species. *P. vulgaris* had a higher anthocyanin content in the seed coat and a higher flavonoid content in the cotyledons than *P. coccineus*, but it did not for the other compounds tested. There was high variability among the accessions that were classified into four phenotypic groups: Two of *P. coccineus*, one of a *P. coccineus–vulgaris* mixed group, and one group of *P. vulgaris.*

## 1. Introduction

The genus *Phaseolus* (Fabaceae) includes more than 400 species, five of which are the best known, including—in order of commercial importance—*Phaseolus vulgaris* L., *Phaseolus coccineus* L., *Phaseolus lunatus* L., *Phaseolus acutifolius* A. Gray., and *Phaseolus dumosus* Macfady. All have their origin, domestication and diversification in America. The first three have a worldwide distribution [1,2,3]. In Mexico and Central America, the greatest diversity of *P. vulgaris* and *P. coccineus* are conserved in situ as part of the traditional cultivation systems, backyards and forests [2]. These regions have the greatest diversity of indigenous groups and ethnolinguistic variants [4,5].

*P. vulgaris* is one of the most documented species of the genus *Phaseolus* in regard to grain composition, including its polyphenol profile [6]; phenols, flavonoids, and carotenes content, as well as their antioxidant activity [7,8,9]; and antinutritional agents, such as trypsin, tannins and lectins [10]. In the case of *P. coccineus*, the concentration of phenolic acids [11], proximal analysis and mineral content have been determined [12,13]. In specific cases, it was observed that the content and composition of *P. vulgaris* grains differ from those of *P. coccineus.* For example, cyanidin-3-glucoside, delphinidin-3-glucoside, cyanidin, methyldelphinidin, and methylcyanidin anthocyanins are more common in *P. coccineus,* among others, whereas peonidin, petunidin and malvidin are more common in *P. vulgaris* [14,15]. This commonality indicates significant differences in grain composition according to the species and genotype.

Wild and cultivated *P. coccineus* are distributed from Mexico to Central America. In Oaxaca, Mexico, both germplasm sources grow simultaneously in adjacent areas; the wild populations grow in natural vegetation zones, fences or back yards near cropping parcels, and farmers commonly move seed from wild populations to cultivated plots, promoting a continual gene flow [16]. Therefore, *P. coccineus* variants preserved on-farm have hybrid combinations of morphological and biochemical traits of seed between wild and cultivated forms. In the evaluation conducted by Quiroz-Sodi et al. [11], they analyzed three cultivated varieties from Queretaro, 650 km away from Oaxaca, but not hybrid combinations or wild forms of *P. coccineus*. Additionally, in the southern region of Mexico, which includes Oaxaca, farmers preserve in situ hundreds of landraces known regionally but not yet evaluated in terms of bioactive compounds, which are broadly used as food by rural communities. In such a communitarian context, it is relevant to evaluate the contribution of *P. coccineus* to the diet in terms of its beneficial potential for health and to compare their composition with *P. vulgaris* throughout the evaluation of secondary metabolites in the cotyledons and seed coat.

Hundreds of *P. coccineus* and *P. vulgaris* landraces are cultivated traditionally in the central-south of Mexico and Central America under environmental conditions restrictive of soil fertility, without the use of agrochemical supplies under rain-fed conditions, and just for the use for self-consumption at the household level; all of these agroecological factors influence grain composition as well as genotype or variant cultivated [17,18]. In addition, in the rural communities from this regions, 65% or more of the population lives in poverty, with a high degree of marginalization, food insecurity, malnutrition associated with excess weight and obesity, and social inequity [19], but they have an annual per capita consumption of beans ranging from 9.8 to 25.9 kg, which implies a high contribution to the communitarian diet [20]. Thus, it is necessary to evaluate the nutritional–nutraceutical contribution between *P. coccineus* and *P. vulgaris* and to obtain estimators of the phenotypic variation inter- and intraspecific in terms of phenolic compounds and antioxidant activity to propose strategies of direct use of such landraces or to start a plant breeding program.

The bioactive compounds identified in the common bean are associated with biological activity in reducing the risk of obesity, diabetes, ischemic cardiomyopathy, cardiovascular diseases, some types of cancer, Alzheimer’s disease, Parkinson’s disease, stress, anxiety, depression, and digestive tract diseases, among others [6,21,22,23,24,25,26]. A similar potential effect can also be attributed to *P. coccineus*, even though it is poorly documented [27]. In particular, the indigenous communities of Mexico and Central America, consume the flowers, green beans (fresh pod) and young shoots of *P. coccineus*, in addition to the grain [28]. In this context, the phenolic compound content and antioxidant activity were tested and compared between landraces of *P. coccineus* and *P. vulgaris* cultivated by indigenous communities of Oaxaca, Mexico.

## 2. Materials and Methods

### 2.1. Germplasm Evaluated

Fourteen native bean populations were evaluated; eight *Phaseolus coccineus* L. (scarlet runner bean) and six *P. vulgaris* (common bean) accessions were collected in different indigenous communities of Oaxaca (13) and Veracruz (1), Mexico (Table 1). After collection, a biophysical description of the seed lot of each accession was made.

### 2.2. Sample Preparation

A sample of 100 g of seeds from each accession was left to soak in distilled water for 12 h at 25 °C, followed by the manual separation of the seed coat from the cotyledons; after this separation, they were handled separately. Subsequently, a 3 g sample of seed coat and another similar portion of cotyledons were homogenized (DAIHAN-brand HG-15-A Gonju-Si, Gangwon, Republic of Korea) with 25 mL of 70% acidified acetone (acetone:water:acetic acid, 70:29.5:0.5, *v*/*v*/*v*) according to the method described by Aquino-Bolaños et al. [29]. Each extract was centrifuged at 4000 rpm for 20 min at 10 °C (Hettich centrifuge, Universal 32R, Tuttlingen, Germany), and the supernatant was recovered. Again, the process was repeated a second time under the same conditions, and, finally, both supernatants were mixed for use in the analyses.

### 2.3. Evaluation of Polyphenols, Flavonoids, Anthocyanins, and Antioxidant Activity

*Total polyphenols.* They were determined by the method described by Singleton and Rossi [30], and the reaction absorbance was measured at 750 nm in a UV-visible spectrophotometer (Jenway 6305, Bibby Scientific Ltd., Dunmow, Essex, UK). The quantification was performed based on a standard curve of gallic acid (0.020 to 0.165 mg mL^−1^), and the results were expressed in mg equivalents of gallic acid per gram of dry sample (mg GAE g^−1^ dw).

*Total flavonoids.* The spectrophotometric evaluation of the total flavonoids was based on the method reported by Zhishen et al. [31]. The absorbance of total flavonoids was measured at 510 nm, and quantification was performed based on a standard curve of (+)catechin (0.012 to 0.121 mg mL^−1^). The values were expressed in mg equivalents of catechin per gram of dry sample (mg CE g^−1^ dw).

*Monomeric anthocyanins.* The anthocyanin content was determined by the differential pH method described by Giusti and Wrolstad [32]. Two dilutions of the extract were made, one with potassium chloride buffer at pH 1.0 and the second with sodium acetate buffer at pH 4.5, diluting each by the previously determined dilution factor. Subsequently, a spectrophotometer was used to generate an absorption spectrum in the range of 460–710 nm to determine the maximum absorbance. The concentration of monomeric anthocyanins (MA) was calculated according to the following equation: AM = (A*PM*FD*1000)/(ε*I), where the absorbance of sample A corresponds to (Aλ510–Aλ700) pH 1.0 − (Aλ510–Aλ700) pH 4.5; MW = 449.2 is the molecular weight of cyanidin-3-glucoside; ε = 26,900 g/mol is the molar absorptivity of the cyanidin-3-glucoside; FD is the dilution factor used; and I is the cell length (1 cm). The results were expressed as mg of cyanidin-3-glucoside per gram of dry sample (mg C3G g^−1^ dw).

*Antioxidant activity.* The antioxidant activity was evaluated by the 2,2-diphenyl-1-picrylhydrazyl (DPPH) method reported by Brand-Williams et al. [33]. A 100 μL sample of the extract was reacted with 2.9 mL of DPPH reagent and allowed to stand for 30 min at room temperature. The absorbance was measured using a UV-vis spectrophotometer (Shimadzu UV-1800, Kyoto, Japan) at 517 nm using 80% (*v*/*v*) methanol as the target. To quantify the antioxidant activity, it was performed based on the inhibition percentage of a standard 6-hydroxy-2,5,7,8-tetramethylchroman-2-carboxylic acid (Trolox) curve in a concentration range of 0.13–0.79 μmol equivalents of Trolox per mL. The results were expressed in micromoles of Trolox equivalents per gram of dry sample (μmol Eq. Trolox g^−1^ dw).

### 2.4. Statistical Analysis

A database with all results was integrated, and an analysis of variance was performed using a completely random design with the nesting of accessions within species. Comparisons among species and accessions were made by the Tukey method (*p* ≤ 0.05). Subsequently, a cluster analysis of hierarchical clustering was performed based on the averages of each variable by accession using the Ward method. Once the groups were defined, a canonical discriminant analysis was performed to test the variability and dispersion of accessions as a function of the phenolic compound content and antioxidant activity. All statistical analyses were performed using SAS software [34].

## 3. Results

According to the description of the seeds by accession, *P. coccineus* presented a variation of 16.2–18.2 mm in length, 9.3–12.9 mm in width, 6.4–8.5 mm in thickness, 14.2–92.0 g in weight, and 62.5–183.5 mL in volume for 100 grains. In the case of *P. vulgaris,* the length, width and thickness of seeds varied from 9.1 to 10.2, from 5.3 to 5.7 and from 3.9 to 4.3 mm, respectively. The weight and volume of 100 grains of *P. vulgaris* ranged from 12.9 to 14.8 g and from 61 to 62.5 mL, respectively. The descriptive statistics showed the substantial differences in the larger dimensions, volume and weight of *P. coccineus* seeds compared to *P. vulgaris*, although the concentration of compounds may have another pattern of variation.

In the analysis of variance, significant differences were determined among species and accessions within species for the polyphenol, flavonoid, and anthocyanin contents, as well as the antioxidant activity by DPPH. Likewise, as a function of the magnitude of the mean squares value, it was estimated that the variance due to species source is greater than the variance among accessions of the same species (Table 2). This indicates that the variation between species is greater than that within each species and reflects part of the evolutionary differences between *P. coccineus* and *P. vulgaris* [35,36].

The average composition and antioxidant activity in the seed coat and cotyledons of *P. coccineus* were significantly different from those of *P. vulgaris*, such as in regard to the polyphenols, flavonoids and antioxidant activity (by DPPH) of the seed coat, and the polyphenols and antioxidant activity of the cotyledons were higher in *P. coccineus* than those in *P. vulgaris*. However, in regard to the monomeric anthocyanin content in the seed coat and flavonoids in the cotyledons, the concentrations were greater in *P. vulgaris* than those in *P. coccineus* (Table 3). In this case, the monomeric anthocyanin content in *P. vulgaris* was nine times higher than in *P. coccineus*, although the latter has a larger seed size and consequently a greater amount of tissue in the seed coat. Additionally, the results show that polyphenol contents, the flavonoid contents, and the antioxidant activity in the seed coat are significantly higher than those in the cotyledons, and, for this reason, the seed coat is now of greater interest as a food supplement and for the processed food industry.

The variation evaluated of phenolic compounds and antioxidant activity within *P. coccineus* and *P. vulgaris* showed different patterns among species. For example, for anthocyanin contents, *P. coccineus* presented a variation of 0.15–1.37 mg C3G g^−1^, and in *P. vulgaris*, the variation was 3.72–9.65 mg C3G g^−1^ in the seed coat. Therefore, depending on the accessions evaluated, the results indicate an intraspecific differentiation in the seed coat anthocyanins within each species. For the polyphenol contents of the seed coat and cotyledons, *P. coccineus* accessions with higher phenol contents in the seed coat also showed high values in the cotyledons, and a similar trend was observed for *P. vulgaris* accessions with a lower phenol content in the seed coat, as they also had a lower content in the cotyledons (Table 3).

For the flavonoid content, four *P. coccineus* accessions stood out with values of 20.2–26.3 mg CE g^−1^ dw in the seed coat and from 0.30 to 0.40 mg CE g^−1^ dw in the cotyledons, which differed from all *P. vulgaris* accessions, as they showed variation between accessions of 10.6–13.7 and 0.30–0.50 mg CE g^−1^ dw of flavonoids in the seed coat and cotyledons, respectively. This outcome indicates that the variability between *P. coccineus* accessions is greater than that between *P. vulgaris* accessions and that there is greater homogeneity in the latter (Table 3).

For the seed coat antioxidant activity by DPPH, it was determined that five *P. coccineus* accessions had the highest values within a range of 1261.0–1789.2 μmol Eq. Trolox g^−1^ dw. In the case of *P. vulgaris,* only one accession had a value of 1132.0 μmol Eq. Trolox g^−1^ dw, while the other accessions showed a variation of 874.2–922.9 μmol Eq. Trolox g^−1^ dw. This same pattern is repeated for the antioxidant activity in the cotyledons; however, the variation between *P. coccineus* accessions was 7.18–13.52 μmol Eq. Trolox g^−1^ dw, and that of *P. vulgaris* was from 6.0 to 8.98 μmol Eq. Trolox g^−1^ dw (Table 3). The results show that the antioxidant activity is significantly higher in the seed coat than that in the cotyledons for both species, and some *P. coccineus* accessions stand out.

**Figure 1:**
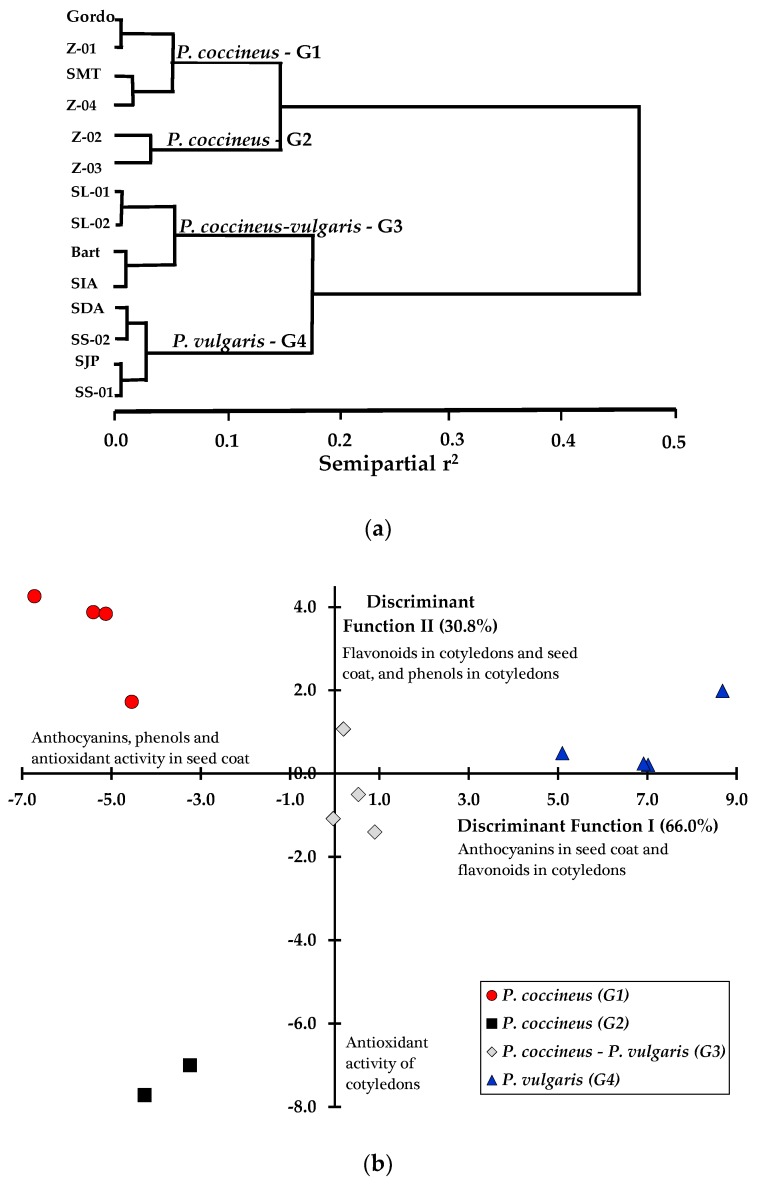
Hierarchical clustering analysis (**a**) and scatterplot of accessions from *P. coccineus* and *P. vulgaris* in the two first discriminant functions (**b**) based on the phenolic compounds and antioxidant activity.

In the clustering and discriminant analysis (Figure 1), the differences and similarities between accessions and species in relation to the polyphenols, flavonoids and anthocyanins content and antioxidant activity in the seed coat and cotyledons were statistically and graphically determined. In the cluster analysis, four contrasting groups were determined: Two *P. coccineus* (G1 and G2) groups, an intermediate group called *P. coccineus–vulgaris* (G3) because it was composed of two *P. coccineus* accessions (SL-01 and SL-02, ID-Accessions) and two *P. vulgaris* accessions (Bart and AIS, ID-Accessions), and a fourth more compact group composed of four *P. vulgaris* accessions (G4: SDA, SS-02, SJP and SS-01, ID-Accessions) (Figure 1a). These results show that there are differences and similarities between *P. coccineus* and *P. vulgaris*, not only in the plant, pod, and seed characteristics but also in the grain composition.

The scatter plot of the discriminant analysis presented in Figure 1b shows that *P. coccineus* accessions are located predominantly on the left side and *P. vulgaris* accessions are on the right side. Thus, four *P. coccineus* accessions (G1), located in the upper left quadrant, differ from the group of *P. coccineus* located in the lower left quadrant (G2); then, there is an intermediate group near the center referred to as the *P. coccineus–vulgaris* combined group (G3), and finally, four *P. vulgaris* accessions (upper right) are preferably associated with a higher anthocyanin content in the seed coat and higher flavonoids in the cotyledons. In contrast, *P. coccineus* showed a lower content of anthocyanins but higher concentrations of phenols and flavonoids, as well as a higher antioxidant activity.

## 4. Discussion

In the general description of seeds, it was determined that *P. coccineus* accessions had significantly greater seed length, width, thickness, weight, density and volume than those of *P. vulgaris* accessions. The different dimensions, among other factors, are due to characteristics inherent to the species and are influenced by agroecological factors, cultivation practices and places of origin [37,38,39]. The accessions used in this work came from the cultivation plots of farmers in Oaxaca and one from Veracruz, Mexico, where the *P. coccineus* variations are highly preferred for the preparation of traditional dishes [40].

The significant differences found between *P. vulgaris* and *P. coccineus* in grain composition (Table 2 and Table 3) suggest that the species have different absorption capacities, translocation and storage of flavonoids, polyphenols and anthocyanins in the cotyledons and seed coat, and these contents are reflected in the antioxidant activity. These interspecies differences were also documented by Onylagha and Islam [41] in regard to the specific contents of flavonoids in the stem and leaves, as well as anthocyanins in the seeds and leaves. In *P. coccineus*, luteolin and epigenin were determined in the stem and leaves, while in *P. vulgaris*, kaempferol and quercetin were identified. In the case of anthocyanins in seeds, delphinidin and cyanidin were identified in *P. vulgaris*, and only cyanidin was identified in *P. coccineus*. The differences between *P. vulgaris* and *P. coccineus* documented by Onylagha and Islam [41] for flavonoids in the stem can explain part of the differences found in our study in respect to the major content of catechins (flavonoids, mg CE g^−1^) and gallic acid (polyphenols, mg GAE g^−1^) in the seed coat of *P. coccineus* relative to *P. vulgaris*. The differences found reinforce the theory that there is an ability to make different nutritional and nutraceutical contributions that are complementary to health through the consumption of *P. vulgaris* and *P. coccineus* because different bioactive compounds are concentrated in the grain.

In both *P. vulgaris* and *P. coccineus,* there was a greater accumulation or concentrations of polyphenols, flavonoids and anthocyanins in the seed coat than in the cotyledons, and, specifically, the amount of anthocyanins in the cotyledons were below the estimated levels. This pattern was also reflected by the greater antioxidant activity in the seed coat than that in the cotyledons (Table 3). Quiroz-Sodi et al. [11] determined a similar trend of a higher polyphenol content in the seed coat than that in the cotyledons for *P. coccineus* and *P. vulgaris*. These trends indicate that the largest amount of bioactive compounds accumulate in the seed coat (e.g., anthocyanins) and not in the cotyledons; in the latter structure, the carbohydrates and protein are accumulated instead [42].

Yoshida et al. [14] and Macz-Pop et al. [15] have documented that different anthocyanins are concentrated in *P. coccineus* grains, and they differ from those found in *P. vulgaris*. Though specific anthocyanins were not tested in this study, it was determined that *P. vulgaris* presents a higher content of anthocyanins in the seed coat than that in *P. coccineus*, a pattern that is repeated with the flavonoid content in the cotyledons. The results are opposite in regard to the polyphenol content and antioxidant activity in the seed coat and grain, as well as the flavonoid content being higher in the seed coat of *P. coccineus* (Table 3). This suggests that *P. coccineus* and *P. vulgaris* should be considered complementary foods, and in the rural communities of Mexico and Central America, they are complemented with the consumption of flowers [28].

Regarding the intraspecific variation of phenolic compounds and antioxidant activity, the results showed high variation in *P. coccineus* and lower variations in *P. vulgaris*. In this case, all *P. vulgaris* accessions visually showed a black seed coat, unlike *P. coccineus* accessions that varied in color from dark-gray to brown and black (Table 1). In the latter case, *P. coccineus* accessions with dark gray, brown, and red seed coats showed a higher phenol content in the seed coat than that in black *P. vulgaris* accessions with the exception of one (SS-02), as seen in Table 3. The same trend was also tested in *P. vulgaris* by Ombra et al. [43] in different traditional varieties from Italy. This finding was reversed for the anthocyanin content in the seed coat; the accessions with higher contents were *P. vulgaris* compared to those of *P. coccineus*, and this indicates that anthocyanins are determinants of the black color in the seed coat.

The SMT, Z-03 and Z-04 accessions of *P. coccineus* stood out for their phenols, flavonoids and antioxidant activity in the seed coat and grain, and in the case of *P. vulgaris,* SS-02 was the best (Table 3). This indicates that within each species, there are accessions that can be used directly to improve the communities’ nutrition or implement a program of genetic improvement towards greater nutritional quality of stand-out grains. That is, few genetic improvement programs of *Phaseolus* have used grain composition as a selection criterion, and the accessions studied here for both *P. coccineus* and *P. vulgaris* are a starting point for a plant breeding program. However, it is necessary to complement them with studies of the amino acid content and other bioactive compounds (e.g., tannins).

The four phenotypic groups classified by the cluster analysis indicate that four *P. vulgaris* accessions were homogeneous, having similarity in their composition, with an intermediate group of *P. coccineus-vulgaris*, and two groups of *P. coccineus* (Figure 1a). The results show a greater dispersion or variability in the phenolic compounds and antioxidant activity between *P. coccineus* accessions than that between *P. vulgaris* accessions, a fact that is confirmed in the scatter plot of the discriminant analysis (Figure 1b), where two distant groups of *P. coccineus*, independent of the *P. vulgaris* accession group, are noted. The diversity evaluated of *P. coccineus* and *P. vulgaris* with respect to seed composition reflects part of the genetic diversity present in Mesoamerica that was evaluated by Sicard et al. [44].

## 5. Conclusions

Based on the community of origin of the accessions and plant material evaluated to determine the phenolic compounds and antioxidant activity, it was concluded that *P. coccineus* and *P. vulgaris* significantly differ in their contents of polyphenols, flavonoids, and anthocyanins, as well as their antioxidant activity in the seed coat and cotyledons. However, both species had greater concentrations in the seed coat than in the cotyledons. *P. vulgaris* had a higher anthocyanin content in the seed coat and a higher content of flavonoids in the cotyledons than that in *P. coccineus*, but it did not have such for the other compounds evaluated. There was high variability between the accessions evaluated, and, specifically, *P. vulgaris* accessions had slightly more than double the anthocyanins compared to those of *P. coccineus*. The variability measured between accessions was classified into four phenotypic groups: Two of *P. coccineus*, one *P. coccineus-vulgaris* combined group, and one group of *P. vulgaris* with greater homogeneity in phenolic compounds and antioxidant activity. Both species are a source of complementary polyphenols, flavonoids and anthocyanins that can be used to improve the diet in rural communities where there is greater access to *P. vulgaris* and *P. coccineus.* Other studies are necessary to determine their potential in preventing some chronic degenerative diseases. Likewise, it is important to promote the cultivation of *P. vulgaris* and *P. coccineus* native species in their place of origin, enhancing them in situ and using them as a cheaper source of food.

## Figures and Tables

**Table 1:** Accessions of *Phaseolus coccineus* and *Phaseolus vulgaris* landraces evaluated.

ID-Accession	Community of Origin (North Latitude; West Longitude; Altitude in Masl ^1^)	Bean Color	Pictures of Grain Characteristic
*Phaseolus coccineus*			
Gordo	Coatepec, Veracruz (19° 27′ 19″; 96° 57′ 31″; 1192)	Light brown	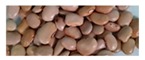
SMT	San Miguel Tlanichico, Zaachila, Oaxaca (16° 55′; 96° 48′; 1520)	Gray-black	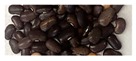
SL-01	Santa Lucía Miahuatlán, Oaxaca (16° 11′; 96° 37′; 2000)	Black	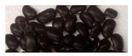
SL-02	Santa Lucía Miahuatlán, Oaxaca (16° 11′; 96° 37′; 2000)	Brown-red	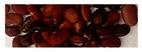
Z-01	Villa de Zaachila, Oaxaca (16° 56′; 96° 45′; 1520)	Red-purple	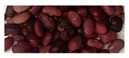
Z-02	Villa de Zaachila, Oaxaca (16° 56′; 96° 45′; 1520)	Gray-black	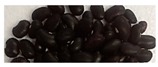
Z-03	Villa de Zaachila, Oaxaca (16° 56′; 96° 45′; 1520)	Gray-black	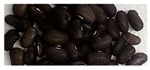
Z-04	Villa de Zaachila, Oaxaca (16° 56′; 96° 45′; 1520)	Gray-black	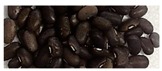
*Phaseolus vulgaris*			
Bart	San Bartolomé Quialana, Oaxaca (16° 54′; 96° 30; 1780)	Black	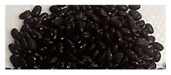
SDA	Santo Domingo Amatlan, Oaxaca (16° 18′; 96° 26′; 1540)	Black	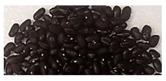
SIA	San Idelfonso Amatlán, Oaxaca (16° 20′; 96° 29′; 1540)	Black	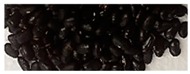
SJP	San José del Peñasco, Oaxaca (16° 18′; 96° 30′; 1589)	Black	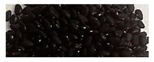
SS-01	San Sebastián Abasolo, Oaxaca (17° 00′; 96° 35′; 1550)	Black	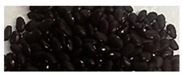
SS-02	San Sebastián Abasolo, Oaxaca (17° 00′; 96° 35′; 1550)	Black	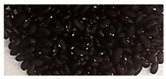

^1^ masl = Meters above sea level.

**Table 2:** Significance of square means of the analysis of variance from phenolic compounds and antioxidant activity of *P. coccineus* and *P. vulgaris* grains.

Sources of Variation	Seed Coat				Cotyledons		
	Total Polyphenols	Flavon.	Anthocyan.	Antioxidant Activity	Total Polyphenols	Flavon.	Antioxidant Activity
Species	31853.3 ** (84.1) ^2^	236.6 ** (65.8)	467.3 ** (98.3)	1563069 ** (85.4)	0.599 ** (79.7)	0.040 ** (64.5)	40.43 ** (77.9)
Accessions (species) ^1^	5999.7 ** (15.8)	122.8 ** (34.2)	8.1 ** (1.7)	267007 ** (14.6)	0.152 ** (20.2)	0.021 ** (33.9)	11.43 ** (22.0)
Error	1.94 (<0.01)	0.05 (<0.01)	0.19 (<0.01)	261.6 (<0.01)	<0.001 (0.1)	<0.001 (1.6)	0.07 (0.1)
Coef. of variation (%)	1.1	1.5	13.6	1.4	1.2	6.6	2.9

Flavon. = Flavonoids; Anthocyan. = Monomeric anthocyanins; ** significant at *p* < 0.01; ^1^ indicates accessions nested in species; ^2^ values in parentheses are percentage variance components.

**Table 3:** Comparison of means among accessions and between species of *P. coccineus* and *P. vulgaris* in relation to the phenols, flavonoids, anthocyanins and antioxidant activity.

ID-Accession	Seed Coat				Cotyledons		
	Total Polyphenols ^1^	Flavon. ^2^	Anthocy. ^3^	Antiox. Activity ^4^	Total Polyphenols ^1^	Flavon. ^2^	Antiox. Activity ^4^
*P. coccineus* (Pc)							
Gordo	152.4 d ^5^	22.0 c	0.15 g	1169.4 d	2.41 b	0.30 d	8.11 f
SMT	218.0 a	26.3 a	0.40 fg	1789.2 a	2.49 a	0.40 b	11.28 b
SL-01	78.3 j	12.7 fg	1.12 fg	888.1 ef	2.18 e	0.30 d	8.40 ef
SL-02	95.5 i	12.1 h	0.30 fg	813.2 g	2.28 d	0.40 b	7.18 g
Z-01	126.5 f	22.7 b	0.52 fg	1261.0 c	2.16 e	0.32 cd	9.08 d
Z-02	136.6 e	6.3 k	1.37 f	1297.4 c	2.08 f	0.20 e	9.96 c
Z-03	194.9 b	9.8 j	0.75 fg	1536.4 b	2.52 a	0.27 d	13.52 a
Z-04	188.8 c	20.2 d	0.67 fg	1498.0 b	2.37 bc	0.40 b	10.00 c
Mean of Pc	148.9 A ^6^	16.5 A	0.65 B	1281.6 A	2.31 A	0.32 B	9.69 A
*P. vulgaris* (Pv)							
Bart	81.7 j	10.6 i	5.12 d	874.2 f	1.86 g	0.30 d	7.41 g
SDA	94.7 i	13.7 e	8.25 b	903.6 ef	2.31 cd	0.50 a	8.44 def
SIA	94.4 i	13.2 ef	3.72 e	905.2 ef	1.81 g	0.30 d	6.00 h
SJP	100.7 h	11.0 i	6.47 c	926.1 e	2.02 f	0.40 b	8.28 f
SS-01	109.3 g	13.1 ef	5.77 cd	922.9 e	2.32 cd	0.40 b	8.98 de
SS-02	123.3 f	12.5 gh	9.65 a	1132.0 d	2.28 d	0.37 bc	8.74 def
Mean of Pv	100.7 B	12.4 B	6.30 A	944.0 B	2.10 B	0.37 A	7.98 B

Flavon. = Flavonoid; Anthocyan = Anthocynin; Antiox. = Antioxidant; ^1^ mg GAE g^−1^ dw; ^2^ mg CE g^−1^ dw; ^3^ mg C3G g^−1^ dw; ^4^ µmol Eq. Trolox g^−1^ dw; ^5^ in column, means with the same letter are not significantly different (Tukey’s test, *p* ≤ 0.05); ^6^ Different capital letters indicate significant differences between species *P. coccineus* (Pc) and *P. vulgaris* (Pv).
